# Smarter Skin Delivery: Nanosomes and Advanced Nanocarriers in Cutting-Edge Cosmetics

**DOI:** 10.3390/molecules31081312

**Published:** 2026-04-17

**Authors:** Barbara Jadach, Zofia Bielawna

**Affiliations:** Device of Industrial Pharmacy, Chair and Department of Pharmaceutical Technology, Poznan University of Medical Sciences, 3 Rokietnicka, 60-806 Poznań, Poland

**Keywords:** nanosomes, liposomes, nanocarriers, nanoemulsions, polymer nanocapsule, skin penetration, cosmetic safety

## Abstract

Nanosomes—lipid vesicles at the nanoscale—enable the encapsulation of both hydrophilic and lipophilic actives and are increasingly used as skin delivery systems in cosmetic products. Alongside nanoemulsions, polymer nanocapsules, and inorganic nanoparticles (e.g., TiO_2_, ZnO, Ag), they can enhance solubility, stability, residence time, and local bioavailability while enabling controlled release. This review summarizes nanocarrier structures, preparation concepts, and skin penetration pathways (transepidermal intercellular/transcellular and transappendageal), and discusses formulation factors that modulate delivery. We highlight applications in UV protection, anti-aging, and fragrance retention, focusing on lipid-based systems (liposomes/nanosomes, ethosomes, niosomes). Safety considerations are critically appraised with reference to EU and FDA frameworks, including physicochemical characterization, dermal penetration, irritation/sensitization, and genotoxicity testing. While most data indicate limited penetration through intact skin for particles ≥20 nm, enhanced uptake may occur under specific conditions (very small size, barrier impairment, mechanical stress), warranting careful risk assessment. We conclude with regulatory and sustainability perspectives and outline research priorities for long-term toxicology, in-use exposure, and standardization of methods.

## 1. Introduction

**Figure 1:**
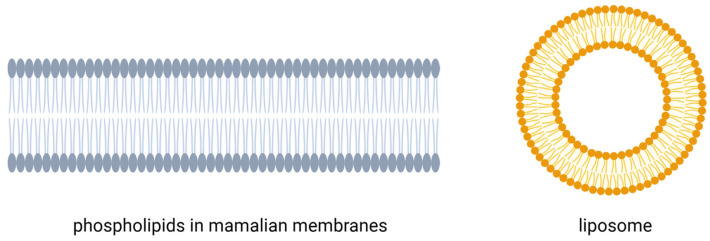
Phospholipid systems in cells and liposome structure (figure is original illustrations created using BioRender. Froelich, A. (2026) https://BioRender.com/4v7l84g; date 13 February 2026).

Nanotechnology has become a significant driving force in the development of modern cosmetic formulations, enabling the creation of nanoscale systems with enhanced physicochemical properties, greater formulation flexibility, and improved delivery of active ingredients. Its essence lies in the manipulation of matter at the nanometer scale, where phenomena unobservable in larger structures occur [1,2,3]. This offers entirely new possibilities for creating materials with unique physicochemical properties. Although the term “nano” has become popular relatively recently, interest in the structure of matter has a long history—from the philosophical works and concepts of Democritus to Richard Feynman’s groundbreaking lecture in 1959, which is often considered the symbolic beginning of the field [2]. The cosmetics industry uses nanocarriers and nanomaterials to improve the bioavailability and effectiveness of active ingredients in cosmetic products. Examples of inorganic nanoparticles commonly used in cosmetic products include gold (Au), silver (Ag), copper (Cu), titanium dioxide (TiO_2_), and zinc oxide (ZnO), inorganic particles characterized by chemical stability and a high safety profile [4,5,6]. Despite the proven positive impact of nanotechnology in various areas of life, the small size of nanoparticles raises much controversy regarding their negative impact on the human body. Their final properties are influenced by their size and the method of preparation. European Union Regulation 1223/2009 states that cosmetics manufacturers are obliged to inform consumers about nanomaterials present in the product’s composition—the prefix “nano” should be clearly visible on the label [7,8]. Among the various nanocarriers utilized in cosmetics, lipid-based vesicles such as liposomes and their nanoscale variants—referred to as nanoliposomes or nanosomes—have attracted particular attention due to their biocompatibility, low toxicity, and structural similarity to biological membranes. In this review, the term nanosomes refers to nanosized liposomal vesicles, typically 30–100 nm in diameter, composed of one or more phospholipid bilayers surrounding an aqueous core. They are produced through conventional liposome fabrication methods followed by size reduction techniques such as extrusion, ultrasonication, or ethanol-based processes. Nanosomes, therefore, represent a size-restricted subclass of liposomes with enhanced surface-to-volume ratio, improved stability, and greater capacity to deliver lipophilic cosmetic actives. The term ‘liposomes’ denotes the broader class of phospholipid vesicles, while ‘nanosomes’ refers specifically to their nanosized variants [9,10]. Their structure, similar to mammalian cell membranes (Figure 1), makes them biocompatible, non-toxic, and biodegradable. Their small size and high surface-to-volume ratio make them superior to conventional liposomes in transporting nonpolar compounds.

Skin, the largest organ of the human body, serves as a natural protective barrier against external factors, including nanomaterials [11,12]. The stratum corneum plays a key role in limiting the penetration of substances, effectively protecting the body from most chemical compounds. Although nanosomes can enhance the delivery of cosmetic actives to the upper layers of the skin, current evidence indicates that, under normal cosmetic use conditions and on intact human skin, nanoscale lipid carriers remain confined to the stratum corneum or hair follicles. Reports of deeper tissue penetration or systemic transport relate to engineered pharmaceutical transdermal systems or to studies using barrier-impaired skin, and therefore cannot be extrapolated to cosmetic applications. At the same time, nanosomes represent only one part of a broader landscape of cosmetic nanocarriers that includes nanoemulsions, nanocapsules, polymeric nanoparticles, metal-oxide nanoparticles (e.g., TiO_2_, ZnO), ethosomes, niosomes, and polymerosomes. Each of these systems exhibits unique structural attributes, preparation strategies, and skin-interaction profiles, which influence their performance, stability, safety, and regulatory status. Therefore, while nanosomes constitute a key focus of this article, their discussion is situated within a wider context of nanotechnology-driven delivery systems currently applied in cosmetic science. Despite growing consumer interest, the introduction of global standards and public education is essential to ensure safe and informed use of nanocosmetics [3,13].

This review aims to summarize the structural characteristics, production principles, and dermal interaction mechanisms of nanosomes and other nanocarriers used in cosmetic products; to evaluate their potential advantages and limitations in topical application; and to examine safety considerations and regulatory requirements governing their use. By integrating mechanistic, technological, and regulatory perspectives, the review provides a comprehensive overview of nanoscale delivery systems in contemporary cosmetology.

## 2. The Origin of “Nano” Ideas

**Figure 2:**
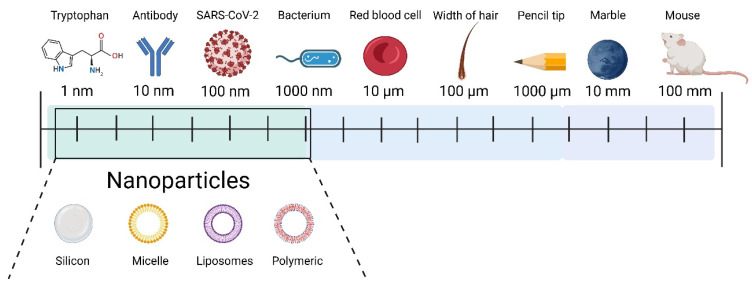
Presentation of scale “nano” (figure is original illustrations created using BioRender. Froelich, A. (2026) https://BioRender.com/7fj1vc9; date: 13 February 2026).

The field of nanotechnology encompasses the creation and use of physical, chemical, and biological systems with structural features between individual atoms or molecules down to submicron dimensions, as well as the assimilation of the resulting nanostructures into larger systems [1]. Its origins date back to 1959, when Richard Feynman, at a meeting of the American Physical Society, posed the question, which roughly translates to: “Why can’t we store all 24 volumes of the Encyclopedia Britannica on the head of a pin?” [2]. Nanoscience (NanoSci) should be distinguished from nanotechnology (NanoTech). Nanoscience studies structures and phenomena on a scale of 1–100 nm, while nanotechnology refers to the practical application of this research, including in the construction of devices [1,2]. To illustrate this scale (Figure 2), the diameter of a human hair is approximately 60,000 nm, and the radius of a DNA double helix is approximately 1 nm.

The origins of considerations about the structure of matter date back to antiquity—as early as the 5th century BC, Democritus postulated the existence of indivisible particles, which we now call atoms [2]. The term nanotechnology refers to any technology operating at the “nano” scale that has real-world applications. This means that it uses individual atoms and molecules to create functional structures [1]. To properly characterize the term “nanotechnology,” it is necessary to define the concept of the “nanoscale,” a scale ranging from 1 to 100 nanometers [1,2,3,14]. A nanometer is a unit of length equivalent to one billionth of a meter (1 nm = 10^−9^ m). Classically, NanoTech encompasses techniques and methods for creating objects in which at least one dimension is in the nanoscale [8,14]. Other definitions of NanoTech include “atomically precise technology” or “atomically precise engineering.” Nanotechnology refers to systems and materials whose structures and components exhibit significantly improved, new chemical, physical, and biological properties due to their nanoscale size [13,14]. The National Nanotechnology Initiative (NNI) in the United States defines nanotechnology as an interdisciplinary field of science, engineering, and technology conducted at a scale of 1–100 nm, where unique phenomena enable new applications in fields such as chemistry, physics, biology, medicine, engineering, and electronics. This definition assumes two basic criteria: scale—encompassing the manipulation of structures in the nanometer range; and novelty—the exploitation of properties characteristic of this scale, unavailable at larger dimensions [1,2,3,13].

### 2.1. Methods of Producing Nanostructures and the Use of Nanotechnology in Everyday Life

**Figure 3:**
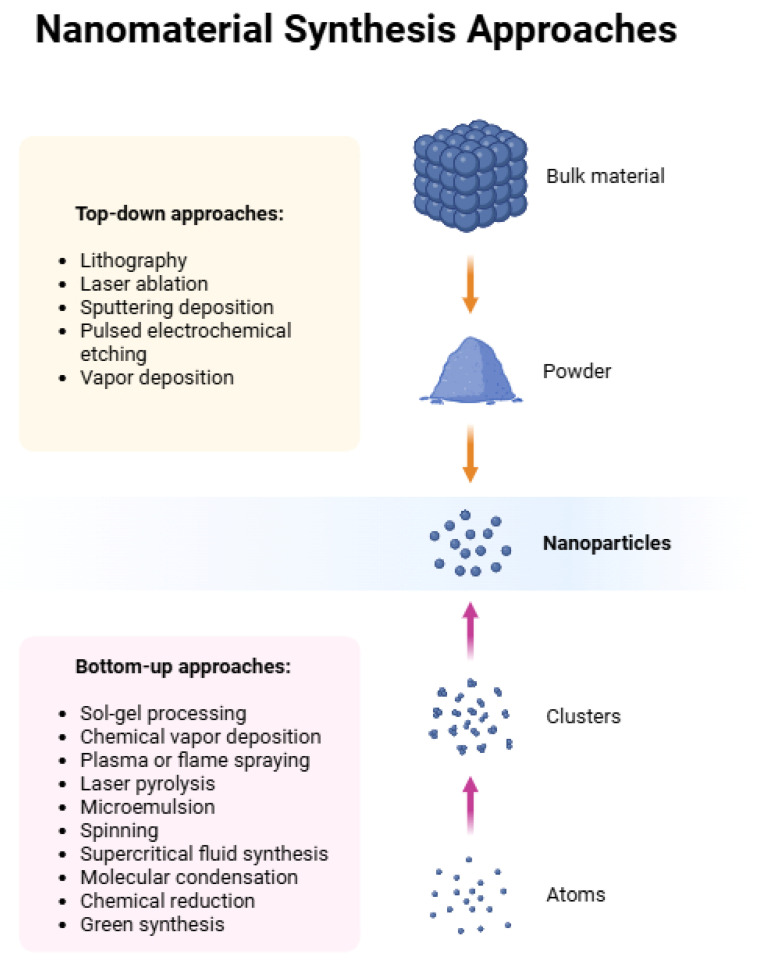
Strategies of preparation of nanomaterials (figure is original illustrations created using BioRender. Froelich, A. (2026) https://BioRender.com/s5zocy3; date 13 February 2026).

Currently, the largest emitters of nanoparticles (NPs) are all technological and chemical processes that utilize high temperatures. Human production of nanostructures utilizes two main types of strategies (Figure 3): bottom-up and top-down strategies [15].

Lipid-based nanocarriers such as liposomes, nanosomes, ethosomes, and niosomes are typically produced using classical vesicle formation techniques. The thin-film hydration method remains the most common approach: phospholipids or surfactants are dissolved in an organic solvent, evaporated to form a lipid film, and hydrated with an aqueous phase containing the active ingredient. Size reduction to obtain nanosomes or nanoliposomes is achieved through probe sonication, extrusion through polycarbonate membranes, or high-pressure homogenization [8,10]. Ethosomes incorporate ethanol in the hydration phase to enhance membrane fluidity, while niosomes are formed using non-ionic surfactants instead of phospholipids, often via heating and hydration or reverse-phase evaporation methods [16]. These preparation strategies determine vesicle size distribution, encapsulation efficiency, and membrane deformability, which affect penetration behavior in cosmetic applications.

Nanoemulsions are produced either by high-energy methods, such as ultrasonication and high-pressure homogenization, or by low-energy methods, including phase inversion temperature (PIT) and spontaneous emulsification [17]. High-energy techniques rely on mechanical forces to break coarse droplets into nanodroplets (<100 nm), while low-energy methods exploit physicochemical transitions in surfactant–oil–water systems to spontaneously generate nanoscale droplets. The choice of method strongly influences droplet size, stability, and suitability for incorporating lipophilic cosmetic actives. Polymeric nanocapsules and nanospheres are typically prepared using nanoprecipitation, interfacial polymerization, or emulsion–diffusion techniques. In nanoprecipitation, a polymer dissolved in an organic solvent is added to an aqueous phase under stirring, resulting in the instantaneous formation of nanostructures as the solvent diffuses. Emulsion-based methods employ surfactants to stabilize droplets within which polymer precipitation or polymerization occurs, encapsulating hydrophobic actives within an oily or polymeric core. The choice of polymer (e.g., PLGA, polycaprolactone) determines biodegradation rate, mechanical stability, and release behavior.

Inorganic nanoparticles used in cosmetic formulations—such as TiO_2_, ZnO, silver, or gold nanoparticles—are commonly synthesized via sol–gel processes, chemical reduction, hydrothermal methods, or controlled precipitation [4,5,6]. In sol–gel synthesis, metal precursors undergo hydrolysis and condensation to form uniform oxide nanoparticles, whereas metallic nanoparticles are frequently produced by reducing metal salts in the presence of stabilizing agents to control nucleation and growth. Particle size and surface chemistry can be tuned through reaction conditions, affecting optical properties, stability, and safety characteristics important for cosmetic applications.

These preparation methods apply not only to inorganic nanoparticles but also to the broader group of nanocarriers discussed in this review. By including lipid-based vesicles, nanoemulsions, and polymeric nanocapsules, the section now reflects the full methodological landscape relevant to nanoscale cosmetic delivery systems.

Among the NPs intentionally produced by humans, we can distinguish carbon nanotubes, fullerenes, nanospheres, nanorods, quantum dots, and nanofibers with a wide range of applications, including controlled release systems for active substances used in dermatology [2,4,18] and other fields of medicine. The importance of NanoTech is constantly growing due to its wide range of applications in various fields of science and technology, such as physics, chemistry, materials science, biology, medicine, management, and the environment [4]. This has led to a close connection between nanotechnology and our daily lives. Nanoscience is a method that is increasingly being used and gaining importance in a wide range of industries [2]. Nanotechnology can play a significant role in expanding innovative methods used to create new products or replace current production equipment. Furthermore, it can contribute to the reformulation of new materials and chemicals to achieve better performance, resulting in reduced material and energy consumption, as well as reduced environmental damage [1,3,13]. One of the most important properties of nanoparticles is their high surface-to-volume ratio [4,13]. This feature is exploited in the production of powerful nanoscale catalysts, which significantly increase the efficiency of chemical reactions and reduce the production of waste materials. Furthermore, the use of NPs in the production of materials can increase their strength while reducing their weight and improving their thermochemical properties. An example of a nanomaterial application is zinc oxide, used in the production of sunglasses. Its use is intended to increase the performance and durability of standard glasses [13].

One of the properties of particles affected by their shrinkage to nanometer sizes is their response to light and electromagnetic waves. Understanding this mechanism has led to the production of nanoadhesives with significant applications in optoelectronics and the electronics industry as a whole [13]. The use of nanomaterials in medicine is quite extensive, ranging from monitoring human heart function to the production of sunscreens with ultraviolet protection using titanium dioxide NPs [4,6,13]. Nanotechnology is also used in the pharmaceutical industry, for example, in drug delivery systems. The creation of nanosized systems is particularly important because it influences certain pharmacokinetic and pharmacodynamic properties, such as solubility, bioavailability, reduced toxicity, and the ability to release drug substances [2,13,14]. In fact, nanosized drugs, due to their large surface areas, demonstrate improved capabilities and performance, including increased solubility, faster therapeutic effects, and, most importantly, lower required doses. This has led to increased efficacy and safety of use, as well as improved patient compliance [13,14,18]. In addition to the significant role of NanoTech in the development of new drug delivery systems (DDS) and formulations, one of the other pharmaceutical fields in which it is used is tissue engineering. This may be due to the exceptional biocompatibility of nanomaterial systems with living cells. Other applications in this field include drug encapsulation, bone replacement (nanohydroxyapatite—“synthetic bone”), prostheses, and implants [18,19,20]. The use of NPs in medicine and pharmacy is influenced by their morphology and shape, which are fully controllable, allowing them to increase drug bioavailability and effectiveness at the sites of action [18,19]. In addition to treating common diseases using nanomedicine systems, pharmaceutical nanotechnology has demonstrated its ability to diagnose and treat diseases such as diabetes and neurodegenerative diseases [1,13]. It has also found applications in information technology and civil engineering, as well as in the oil, gas, and petrochemical industries—in this case, for the production of nanocoatings resistant to heat, corrosion, abrasion, and friction [13]. Having outlined the general role of nanotechnology in modern formulations, the following section focuses specifically on how nanoscale systems are applied within cosmetic products and how their structural diversity determines functional performance.

### 2.2. Use of Nanotechnology in Cosmetics

The cosmetics industry utilizes nanocarriers and selected nanoparticles to improve the bioavailability and effectiveness of active ingredients used in cosmetic products such as sunscreens, anti-aging creams, moisturizers, and fragrances [4,14,15,21]. Several delivery methods for NPs are used in cosmetic formulations due to the diversity of ingredients. This aids in the effective delivery of active ingredients to the skin [5,8,15,21]. This improved delivery results from enhanced thermodynamic activity and increased availability of the released active ingredient at the skin surface, not from deep penetration of intact nanocarriers.

**Figure 4:**
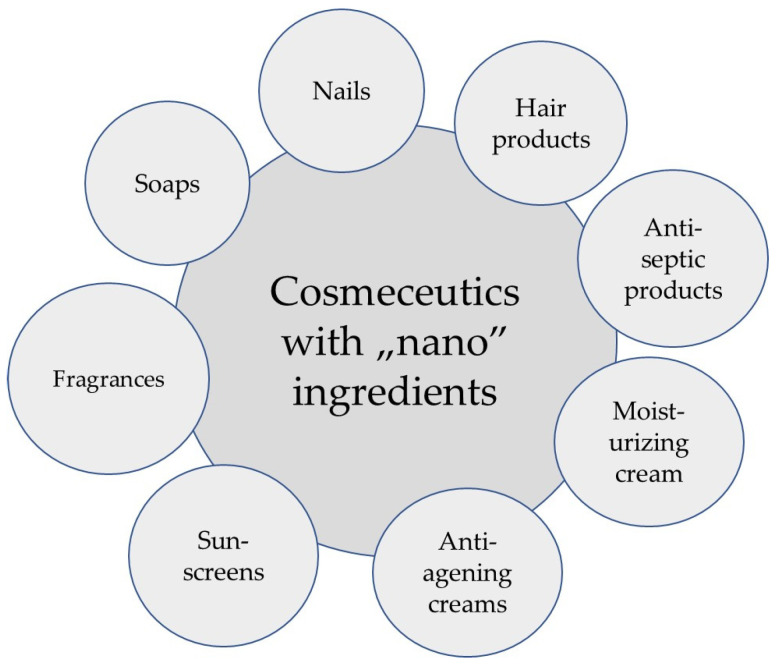
Types of cosmetic products prepared with the use of NanoTech (figure prepared by authors).

Products formulated with nanotechnology (Figure 4) facilitate rapid and effective absorption of ingredients through the skin [4,21]. Active ingredients are adsorbed onto the surface of nanocarriers (e.g., nanoemulsions, nanocapsules, liposomes), which act as carriers. To optimize the benefits of the activities dependent on the shape and size of nanoparticles and improve the performance of cosmetic products, cosmetics may contain NPs of varying morphology and chemical composition. Various metal and metal oxide NPs are used in the production of cosmetic products, such as titanium dioxide, gold, silver, iron oxide, zinc oxide, and carbon-based particles [4,5,6]. These substances can act as carriers of active ingredients in cosmetic products, as well as texture-enhancing ingredients or as active ingredients themselves, increasing the effectiveness of cosmetic products. Furthermore, due to their extensive antibacterial properties and ability to act as preservatives, silver NPs are widely used in cosmetic formulations. For example, products containing silver nanoparticles are used to disinfect skin cuts [22,23]. Each type of nanomaterial has its own unique properties and potential applications. We distinguish the following materials: nanocapsules, dendrimers, nanoemulsions, nanoliposomes, and nanohydroxyapatite materials. They are used in cosmetic products, including oral care products, as well as in facial and body care products, as carriers for active ingredients [13,14,15,20,21].

#### 2.2.1. Classification of Nanomaterials Used in Cosmetic Products

**Figure 5:**
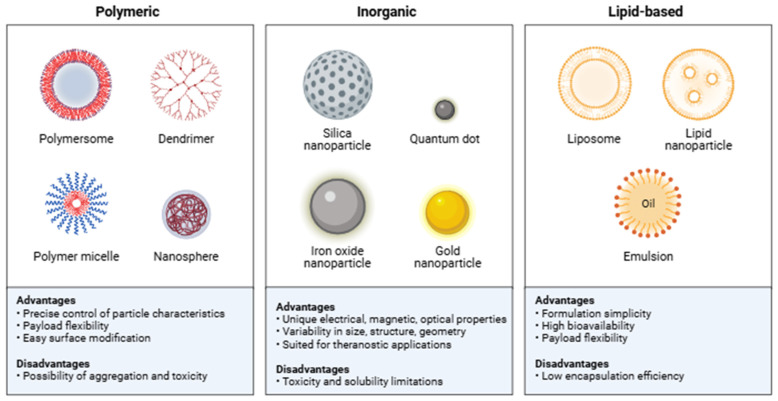
Examples of nanoparticles (figure is original illustrations created using BioRender. Froelich, A. (2026) https://BioRender.com/oik0899; date 13 February 2026).

Nanomaterials are classified in various ways (Figure 5, Table 1). One of the most widely used divisions is into organic and inorganic nanostructures [4,8,21]. We also distinguish between the method of construction, i.e., the way in which the incorporated substance is placed. When the substance is incorporated into the core in a solid form, we are dealing with a core/shell structure. However, when we want to incorporate a liquid substance, we speak of a yolk/shell structure, i.e., a “liquid core/shell.” These structures are designed to control the release or delivery of active substances in the cosmetic [3,4,15,21]. Nanomaterials can occur in various shapes, such as spheres (e.g., carbon fullerenes), tubes (e.g., carbon nanotubes), or dendritic forms (e.g., titanium dioxide particles) [4,13,19]. The term nanoparticle typically refers to solid colloidal carriers with predominantly spherical morphology, often produced from polymers, lipids, or inorganic materials. Other morphologies, such as nanotubes, nanosheets, nanofibers, or dendritic nanostructures, fall under the broader category of nanomaterials or nanostructured materials, but are not strictly classified as nanoparticles in the pharmaceutical sense. Titanium dioxide particles, depending on their crystalline form and production method, may present irregular or dendritic structures, but they are not an example of “dendritic nanoparticles” in the pharmaceutical definition. Inorganic nanoparticles are distinguished by their chemical stability and high safety profile, although this requires individual assessment depending on the type of nanomaterial.

Understanding the structural and physicochemical properties of each nanocarrier type is essential, as these characteristics directly determine their behavior in cosmetic formulations and define their potential applications.

Throughout this review, the term ‘enhanced penetration’ refers to improved solubilization, follicular deposition, thermodynamic activity, or partitioning of the released active ingredient, rather than the movement of intact nanocarriers into viable skin layers. Although nanosomes represent a nanoscale subset of liposomes, several functional features justify their distinct consideration in cosmetic delivery systems. Their reduced diameter (typically 30–100 nm) not only increases the surface-to-volume ratio but also enhances vesicle uniformity, contributing to more predictable encapsulation behavior and improved dispersion stability in fluid or semi-solid formulations [9,10,24]. Compared with conventional liposomes (>100 nm), nanosomes show superior ability to accumulate within hair follicles, an advantageous reservoir site for cosmetic actives, owing to size-dependent follicular targeting effects reported for nanoscale carriers [25]. In contrast to ethosomes, which rely on ethanol-induced membrane fluidization but often exhibit lower formulation stability, nanosomes maintain structural integrity without requiring high ethanol content, increasing their compatibility with sensitive cosmetic ingredients [21]. Transferosomes offer ultradeformability but are limited by scalability and long-term stability constraints in commercial formulations. Nanosomes, therefore, occupy a favorable middle ground: they are more stable than ethosomes and more industrially feasible than transferosomes, while providing greater surface-area-driven release efficiency and better follicular localization than larger liposomes. These attributes, supported by literature on cosmetic delivery, highlight nanosomes as a particularly versatile and commercially relevant platform within the broader family of lipid-based nanocarriers [3,21]. Beyond physicochemical and penetration-related properties, practical factors such as industrial scalability, production cost, and technology readiness level (TRL) critically influence the feasibility of nanocarrier implementation in cosmetic formulations. Nanoemulsions and niosomes exhibit the highest industrial scalability due to robust, low-cost manufacturing processes, whereas transferosomes and polymerosomes remain limited by stability constraints and complex production steps. Lipid-based nanosomes and conventional liposomes occupy an intermediate position, combining manufacturability with established commercial maturity. Polymeric nanocapsules, while offering excellent stability and controlled release, require more resource-intensive fabrication, resulting in higher production costs and lower TRL in cosmetics compared to pharmaceutical applications [26,27]. These considerations highlight that the suitability of each nanocarrier system depends not only on functional performance, but also on practical manufacturing and commercialization factors relevant to large-scale cosmetic production. Overall, nanosomes represent a balanced and versatile nanocarrier system, combining biocompatibility, encapsulation efficiency, stability, and regulatory acceptance, which justifies their particular emphasis in this review. The comparative table presented above also functions as a conceptual schematic, summarizing key performance-defining attributes across nanocarrier classes, including composition, stability, penetration tendencies, safety parameters, and technology maturity. Stability profiles further differentiate nanocarriers: conventional liposomes are prone to phospholipid oxidation and aggregation over time; nanosomes show greater colloidal stability due to smaller hydrodynamic diameters; ethosomes demonstrate reduced long-term stability because of ethanol-driven bilayer disorder; nanoemulsions offer excellent kinetic stability but may undergo Ostwald ripening at elevated temperatures; polymeric nanocapsules and polymerosomes show the highest resistance to hydrolytic and mechanical stress [9,10,24]. These stability trends determine not only shelf-life but also the feasibility of integrating specific nanocarriers into complex cosmetic bases. The normal size of metal particles ranges from 1.5 to 5 nm at a concentration of 10 ppm. Examples of inorganic NPs used in widely available cosmetic products include gold (Au), silver (Ag), copper (Cu), titanium dioxide (TiO_2_), and zinc oxide (ZnO) [4,5,6]. Nanoparticle titanium dioxide has found its use in the production of sunscreens due to its ability to absorb ultraviolet (UV) radiation [5,6]. In terms of chemical properties, it is a white, chemically inert powder with exceptional durability. Its precise properties depend on the degree of fineness. Unlike other physical sunscreens, which tend to whiten the skin after application, resulting in increased susceptibility to abrasion and, consequently, reduced protective properties, nanoparticle titanium dioxide is a transparent substance [26,27]. This not only improves the product’s structure but also improves comfort of use without compromising photoprotective properties. According to various reports, nano-titanium dioxide used in UV-protective products can also neutralize free oxygen radicals [27,28]. Titanium dioxide’s UV-protective properties have also been utilized in the coatings and paint industry, where it is often used as a pigment. In medicine, it is a component of products for disinfecting surgical instruments and various surfaces [4]. The regulatory status of titanium dioxide (TiO_2_) varies across application fields. In the European Union, TiO_2_ has been banned as a food additive due to uncertainties regarding its genotoxic potential, while in pharmaceuticals, it is undergoing progressive substitution despite remaining temporarily permitted as an excipient. In cosmetics, TiO_2_ continues to be allowed both in pigment and nanoform; however, its use is subject to strict conditions. In particular, nano-TiO_2_ cannot be incorporated into products that may lead to inhalation exposure, such as sprays or loose powders, due to the inhalation-related risks identified by regulatory authorities. Cosmetic ingredients containing TiO_2_ in nanoform must also be clearly labeled with the ‘[nano]’ suffix in the INCI list. These distinctions highlight the importance of route-specific safety assessment when evaluating TiO_2_ in cosmetic formulations [26,27]. Colloidal gold (nanogold) exhibits structural variability—its particles can occur in the form of nanocubes, nanostars, nanorods, and other shapes. Its remarkable skin-regenerating properties make it widely used in active inflammatory conditions, various hypersensitivities (including those to other cosmetic ingredients), and sunburn. Furthermore, nanogold particles have collagen-stimulating properties [29]. Silver nanoparticles exhibit antiseptic, disinfectant, and antibacterial properties. Consequently, they are used in cosmetic products such as antiperspirants, shampoos, makeup removers, and foot creams. The particle size of nanosilver used in cosmetics ranges from 1 to 100 nm. Colloidal nanosilver is most often found in preparations for various skin conditions, including psoriasis and acne, as well as for difficult-to-heal wounds [4,30]. Other nanomaterials, such as polymer nanocomposites, have found applications in cosmetic packaging due to their increased mechanical strength, resistance to temperature changes, and hardness. The use of nanocomposites will result in packaging with improved durability, which will also extend the shelf life of cosmetic products. The structure of polymer nanocomposites is biphasic—the polymer shell contains evenly distributed filler particles. Compounds used to produce nanocomposites include cellulose nanoparticles, layered silicates, carbon nanotubes, and chitin and silica nanoparticles [30,31,32]. Based on a report published by Vision Research Reports, the value of the international nanomaterials market in 2019 was estimated at approximately USD 8.5 billion, with expected growth in the coming years [4,30]. In cosmetic products, in addition to inorganic/metallic nanoparticles, we can also observe the presence of lipid nanocarriers or liposomes. The size of such NPs determines their potential use, for example, as a sorption promoter or as a substance increasing the bioavailability of a given cosmetic ingredient. Furthermore, particle size influences the aesthetics of a cosmetic product and ease of application [4]. NPs are used externally in cosmetic products for skin, nail, hair, and lip care. A nanoemulsion (NEm) is a type of emulsion in which liquid droplets with a diameter below 100 nm are uniformly dispersed within the continuous phase [17,21,33]. These are colloidal dispersions that can be used as carriers for active substances with limited water solubility [20]. NEm typically consist of oil nanodroplets dispersed in water or water nanodroplets dispersed in oil. Surfactants are used to increase their stability [20,21]. A key limitation of these nanocarriers is their thermodynamic instability when their size exceeds 500 nm. This results in complete light penetration through the emulsion. The use of NEm in a product improves its ease of application due to its exceptionally light formula [17,21]. Furthermore, nanoemulsions improve the interaction between the skin and the emulsion, which allows more active substances to come into direct contact with the skin surface [20]. The fine nanoemulsion droplets do not clog pores, allowing for the free flow of air and water. Due to their lipophilic structure, NEms are better at enhancing the availability and diffusion of lipophilic substances within the stratum corneum than conventional liposomes, primarily due to improved solubilization and partitioning, not deeper penetration of intact droplets. Furthermore, they demonstrate bioactivity, supporting the skin’s barrier function by reducing transepidermal water loss. Considering the behavior of nanocarriers and nanoparticles contained in cosmetics, these droplets enhance the delivery of active ingredients primarily through improved solubilization, follicular deposition, and partitioning into skin lipids, rather than through penetration of intact droplets through channels between stratum corneum cells, i.e., corneocytes, without requiring penetration promoters or modification of the outer layer of the epidermis. In cosmetic applications, this ‘enhanced penetration’ reflects primarily improved solubilization, skin deposition, and partitioning of the released active, rather than translocation of intact nanoemulsion droplets. They operate by creating microchannels that allow the penetration of larger particles. They can also act as a “reservoir” for active substances previously applied to the skin. NEms used in cosmetic preparations utilize oxyalkylene glycols and some vegetable oils. Protein polymers with attached aromatic hydroxyl groups have been used in nail polishes. They provide good shine, water resistance, and also increase the viscosity and strength of the composition [10]. Topical preparations are typically oil-in-water emulsions. The oil phases are typically of natural origin, such as sunflower oil, tea tree oil, soy lecithin, olive oil, or cosmetic oils. NEms also contain surfactants, usually nonionic, such as Tween 20, Tween 80, polyvinyl alcohol, or natural products such as sucrose esters. A common additive that facilitates the application of cosmetic products to the skin is a cross-linking agent, which transforms the formulation into a gel. Examples of such cross-linking agents include Carbopol 940, glycerol, or PEG. Nanoemulsions containing tea tree oil are used, among other things, for the transdermal delivery of fish protein hydrolysates (FPH). Other NEms based on Compritol ATO with a surfactant mixture have been used as resveratrol carriers for dermal application. They ensured high drug concentration and effective action: antioxidant, anti-inflammatory, and anti-wrinkle after exposure to UVB radiation [34].

Nanocapsules (NCap) are nanostructures made of polymeric units dispersed in an aqueous or oily phase. These formulations are considered excellent carriers for certain sensitive active substances, such as vitamin D or potent cosmetic active ingredients [29]. NCap are used in cosmetics for purposes such as masking undesirable odors, increasing the stability of active substances, and minimizing incompatibilities between ingredients in finished formulations. Polymer NCap can be applied directly to the skin as a suspension or integrated into semi-solid systems as nanocarriers. The skin penetration rate of these structures can be precisely controlled by modifying the surfactant-to-polymer ratio during the synthesis stage. In cosmetic contexts, this enhanced delivery reflects controlled release and improved diffusion of the active ingredient, not deep penetration of the nanocapsule itself. Polymer NCap, designed to encapsulate natural plant extracts and vitamins, have also been produced and then incorporated into semi-solid formulations such as creams, lotions, and moisturizers. After application, these formulations are activated by factors present in damaged skin (e.g., enzymes, pH changes), initiating the controlled release of active substances in selected skin layers. Furthermore, the use of polymer nanocarriers effectively extends fragrance longevity by encapsulating fragrance molecules [35]. NCap exhibit several advantageous properties compared to nanospheres, particularly in their ability to encapsulate natural products within an oily core that acts as a protective barrier, shielding the encapsulated natural products from the external environment, thereby increasing stability and preventing degradation [19]. Encapsulating lipophilic ingredients in rigid NCpa, such as polycaprolactone capsules, has been shown to slightly increase product penetration through the skin and also enhance UV protection, allowing for the use of lower concentrations of cosmetic sunscreen. With the exception of sunscreens and after-sun cosmetics, which can be applied to sunburned skin, unlike topical dermatological medications, cosmetics are intended exclusively for use on healthy skin. This is reflected in EU guidelines, which require that safety tests (e.g., irritation, penetration, sensitization) be conducted on healthy skin, not on damaged or diseased skin [30]. Gold particles are also an interesting example of nanomaterials, including gold nanospheres, gold nanotubes, hollow gold nanospheres, gold nanostars, gold nanocages, and gold rings. Hollow gold nanospheres have attracted considerable attention due to their excellent physicochemical properties and low toxicity. This hollow structure generates minimal mass in the same size as gold nanostructures of various morphologies, enabling them to load drugs and other functional materials, which offers significant advantages in the field of drug and other substance delivery. Furthermore, thanks to a number of advantages, such as good biocompatibility, excellent photothermal conversion, and easy modification with various biological molecules, hollow gold nanospheres have broad application prospects in the field of biomedicine [36]. While nanocarriers offer clear advantages in cosmetic performance, their interaction with the skin barrier also raises important safety considerations that must be evaluated in parallel with their functional benefits. These functional advantages must, however, be considered alongside the safety implications associated with nanoscale materials, particularly regarding their interaction with the skin barrier and regulatory oversight.

#### 2.2.2. Safety of Nanotechnology in Cosmetics

**Figure 6:**
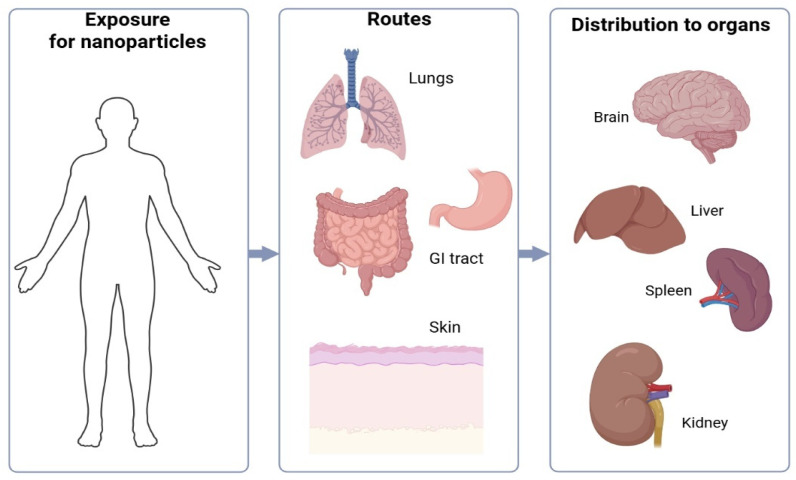
Routes of exposure of nanoparticles and main organs for cumulation (figure is original illustrations created using BioRender. Froelich, A. (2026) https://BioRender.com/bsdgjmw; date 13 February 2026).

Many studies demonstrate the positive impact of nanotechnology in various areas of life. However, the small size of nanoparticles raises considerable controversy regarding their negative impact on the human body. Figure 6 presents possible places in the human body that can accumulate nanoparticles. Their final properties are influenced by their size and the method of production. It has been proven that the top-down method can unintentionally release solvents or reducing agents used during production into the environment [13]. Particles measuring 10–50 nm can reach the lungs. Controversial ingredients include carbon black, used in mascaras, and nanosilica. A study conducted on mice demonstrated that nanoparticles delivered to the body can penetrate the blood–brain barrier. The study used 50 nm particles. Exposure to the nanoparticles occurred for 4 weeks, 5 times a week, for 4 h a day. After this period, the presence of nanoparticles was demonstrated in the spleen, liver, testes, lungs, brain, and, in smaller amounts, in the heart and kidneys. Furthermore, in 2007, a thesis was developed that the particle size is so small that it allows penetration into the cell, simultaneously altering its functionality. Therefore, it is recommended to avoid using preparations containing nanoparticles on damaged skin. Studies on the use of nanoparticles as sunscreens have shown that titanium dioxide in this form tends to accumulate in the epidermis, specifically in its stratum corneum [26,27,28].

When TiO_2_ was applied to sunburned skin, a slight increase in NP concentration was observed in the stratum corneum, but no penetration into the deeper layers was observed. Apart from studies on the effects of titanium dioxide on the body, data on the toxicity of such small particles in cosmetic products is relatively scarce. Therefore, studies are necessary to determine the toxicity for consumers and those working in the production of these nanomaterials, as well as their environmental impact [4]. In addition to inorganic nanoparticles, cosmetic formulations frequently employ a wide range of organic nanocarriers—such as nanoemulsions, lipid-based vesicles (including liposomes, nanosomes, ethosomes, and niosomes), and polymeric nanocapsules—which exhibit distinct safety profiles. These systems differ fundamentally from solid nanoparticles in their composition, degradation behavior, and interaction with the skin barrier. Lipid-based carriers are generally recognized as highly biocompatible due to their structural similarity to biological membranes, although their safety must still be evaluated with regard to surfactant content, vesicle stability, and potential irritation. Nanoemulsions, while not composed of solid particles, require assessment of surfactant levels and droplet stability, as destabilization may influence penetration behavior. Polymeric nanocapsules depend on the biodegradability and purity of the polymer matrix, which determines their accumulation potential and degradation products. As such, safety evaluation of nanocarriers must consider the specific material class, physicochemical properties, exposure route, and intended use, rather than applying conclusions derived solely from inorganic nanoparticle studies. According to European Union Regulation 1223/2009, cosmetics manufacturers are obliged to inform consumers about nanomaterials contained in the product, and the prefix “nano” should be clearly visible on the label [4]. This document provides a strong, universally recognized system that establishes product safety, taking into account the latest scientific data, including the use of nanomaterials [3]. Given that the safety of nanomaterials depends not only on their intrinsic properties but also on exposure route and formulation context, regulatory frameworks play a pivotal role in ensuring their responsible and controlled use in cosmetics [3,7]. According to Regulation (EC) No 1223/2009, a ‘nanomaterial’ is defined as an insoluble or bio-persistent and intentionally manufactured material with one or more external dimensions, or an internal structure, on the scale of 1–100 nm. Cosmetic ingredients present in nanoform must be clearly identified in the product’s INCI list with the suffix ‘[nano]’. Nanomaterials used in cosmetics are also subject to pre-market notification through the Cosmetic Products Notification Portal (CPNP) [7,8]. The Scientific Committee on Consumer Safety (SCCS) has issued several opinions relevant to nanomaterials commonly used in cosmetic products. For titanium dioxide (TiO_2_) and zinc oxide (ZnO), the SCCS concluded that their nanoforms are safe for use as UV filters in dermal applications, provided that particles are not inhaled and do not penetrate viable skin layers. Both materials show minimal systemic uptake when applied to intact skin. However, inhalation of respirable TiO_2_ or ZnO particles poses potential health risks, and the EU prohibits their use in cosmetic products that may lead to aerosolization or inhalation exposure, such as sprays or loose powders [26,27]. In addition to cosmetic-specific legislation, nanomaterials fall under the EU REACH Regulation, which requires substance registration, characterization, and safety assessment at the nanoform level [8]. Recent REACH updates clarify that manufacturers must provide detailed physicochemical data—including particle size distribution, shape, surface area, and surface chemistry—for each nanoform placed on the market. Together, these regulatory frameworks ensure that nanomaterials used in cosmetic formulations undergo rigorous safety review, with explicit differentiation between dermal and inhalation exposure pathways. This structured overview outlines the key regulatory steps applied to cosmetic nanomaterials in the EU, functionally corresponding to a regulatory evaluation workflow. This structured overview functions as a regulatory workflow in text form, mapping the sequence of EU regulatory requirements—from legal definition and INCI labeling, through SCCS evaluation and CPNP notification, to REACH nanoform-specific data obligations [8]. The Scientific Committee for Consumer Affairs, based on research and scientific publications, states that there is no clear evidence that NPs with a diameter of 20 nm or larger can penetrate intact skin into living cells. However, there is already evidence that NPs with diameters smaller than 10 nm can penetrate through the skin into living tissues (into the stratum spinosum of the epidermis and even into the dermis). However, the above claims only apply to healthy, intact skin. In the case of skin affected by various diseases, such as psoriasis or atopic dermatitis, there is no significant information regarding nanoparticle penetration. A similar situation occurs in the case of skin damage, for example, from sunburn. All these abnormalities lead to damage to the skin barrier, which facilitates the penetration of nanostructures. Other activities that affect transdermal penetration include rubbing, bending, and other mechanical actions [4].

Although the stratum corneum constitutes the major rate-limiting barrier to nanoparticle penetration, its integrity is notably altered in dermatological conditions such as psoriasis or atopic dermatitis. These disorders are characterized by disrupted lipid lamellae, increased transepidermal water loss, and impaired cohesion between corneocytes, which collectively result in increased permeability of the skin barrier [11,12]. Such structural changes may facilitate the passage of smaller nanomaterials or highly flexible nanocarriers; however, the available data remain limited, heterogeneous, and often dependent on the specific nanomaterial tested, the experimental model used, and the severity of the disease [14,18,20]. Current studies suggest that penetration tendencies may increase in compromised skin, but controlled in vivo investigations in humans are scarce, and no definitive conclusions regarding systemic uptake can be drawn. This knowledge gap highlights the need for further research to characterize nanoparticle behavior under inflammatory or barrier-disrupted conditions, particularly in the context of cosmetic applications, where products are intended exclusively for use on healthy skin. Interpretation of dermal penetration studies requires careful consideration of the experimental model. In vitro assays and reconstructed skin equivalents provide mechanistic insight but often overestimate permeability due to incomplete barrier maturation [20]. Ex vivo human or animal skin maintains structural integrity but lacks perfusion, metabolism, and immune reactivity, which limits extrapolation to in vivo conditions [18,19]. Animal studies enable whole-organism evaluation; however, animal skin typically exhibits higher permeability than human skin. Consequently, human in vivo studies represent the highest level of evidence for cosmetic risk assessment, consistently showing that most nanoparticles ≥ 20 nm remain confined to the superficial stratum corneum or follicular openings [26,27,30]. The manuscript now differentiates these evidence levels to provide a clearer and more robust interpretation of nanocarrier behavior. Safety assessment of cosmetic nanomaterials is guided by standardized international methodologies, in particular the OECD Test Guidelines (TGs) that form the basis of non-animal safety evaluation. Skin irritation is commonly assessed using reconstructed human epidermis (RHE) models—such as EpiDerm™, EPISKIN™, and SkinEthic™—which are validated under OECD TG 439 [3,21]. Skin sensitization is evaluated using OECD TG 442C (DPRA), TG 442D (KeratinoSens™), and TG 442E (h-CLAT), which together form an integrated non-animal testing strategy for identifying potential sensitizers [20]. Dermal absorption studies follow OECD TG 428 [26,27], which provides a harmonized protocol for assessing permeation using human or animal skin ex vivo, while eye irritation potential can be evaluated using OECD TG 492 [30]. These guidelines, together with physicochemical characterization requirements, enable structured and internationally recognized safety evaluation of nanomaterials used in cosmetics. According to the FDA (Food and Drug Administration), for any cosmetic product with new or modified properties, data needs and testing methods should be assessed to address any unique properties and functions of nanomaterials used in cosmetic products. The FDA recommends that the safety assessment of cosmetic products using nanomaterials consider several important factors, including physicochemical characteristics, agglomeration and size distribution of nanomaterials under toxicity testing conditions and as expected in the final product, contaminants, potential routes of exposure to nanomaterials, in vitro and in vivo toxicological data on nanomaterial components and their contaminants, dermal penetration, potential inhalation, skin and eye irritation, sensitization studies, and genotoxicity studies [3]. If a manufacturer wishes to use a nanomaterial in a cosmetic product, either a new material or a modified version of a previously marketed ingredient, the guidelines recommend that the manufacturer meet with the FDA to discuss the test methods and data needed to support the product’s safety, including short-term toxicity and other long-term toxicity data [3]. Beyond the current regulatory guidelines, the future development of cosmetic nanomaterials will depend on addressing emerging scientific, technical, and societal challenges that shape the trajectory of the field.

### 2.3. Cosmetic Formulations Based on Nanotechnology

Nanotechnology enables the use of advanced methods for delivering active ingredients, such as nanoemulsions, nanocapsules, and nanosomes. This allows the creation of nanocosmetics with formulations tailored to specific skin needs. Nanocosmetics are cosmetic products utilizing nanotechnology, based on the modification of materials with dimensions below 100 nanometers. NPs, thanks to their flexibility and adaptability, allow for the development of solutions that support, among other things, the fight against discoloration, dryness, and signs of aging. Often, a single product combines several skincare functions [21]. Benefits are summarized in Table 2.

#### 2.3.1. UV Protection Products

Polymer, metallic, and fullerene NPs are used to protect against ultraviolet (UV) radiation. Polymer nanoparticles, including Benzophenone-3, are a molecular filter that protects cosmetics from deterioration due to light exposure and protects the skin from the harmful effects of solar radiation. This ingredient is listed as a radioprotectant and generally poses no health risk, except for its potential for allergic and photoallergic reactions. Nanoencapsulation of traditional UV filters in organic nanocarriers, such as biocompatible and biodegradable polymers: poly(lactic acid), poly(glycolic acid), their copolymer PLGA, poly(caprolactone), N-(2-hydroxypropyl)-methacrylate copolymers, and poly(amino acids), improves their retention in the skin, stability, and radiation-blocking effect. Poly(capronolactone) particles are particularly preferred as nanocarriers because they ensure reduced skin penetration, allowing them to retain the preparation on the skin surface for a long time without penetrating deeper layers [34]. Metallic TiO_2_ and ZnO nanoparticles have been known for decades as effective UV filters [29,30]. However, their use is limited because they also reflect light in the visible spectrum, creating an undesirable white, granular coating on the skin surface. These metal oxide nanoparticles, measuring 40–60 nm, are more effective and transparent due to their absorption, reflection, and scattering of UV light, making them acceptable to consumers and suitable for use in sunscreen products. Fullerenes (compounds containing 60 carbon atoms-C60) are known as free radical scavengers. One C60 molecule neutralizes as many as 34 methyl radicals [34].

#### 2.3.2. Anti-Aging Products

Nanoscale delivery systems (nanocarriers and nanoparticles) are used as functional cosmeceutical systems to slow down aging and protect the skin from external stress factors such as ultraviolet radiation and pollutants. Biodegradable nanocarriers (e.g., polymeric nanoparticles, nanocapsules) are suitable for encapsulating active ingredients, allowing for sustained release and thus a prolonged therapeutic effect [34]. Non-degradable nanoparticles, such as TiO_2_ and ZnO, protect the skin from photoaging [30,34]. Currently, the cosmetics industry utilizes less toxic, biocompatible NPs such as silver nanoparticles, platinum-palladium nanoparticles, and gold NPs, which possess anti-wrinkle, brightening, or antioxidant properties thanks to strong reducing agents such as rutin and compounds derived from the raw material Panax ginseng. Surface modification of NPs is also possible, expanding their range of applications [34]. The leading delivery systems for anti-aging active ingredients to the skin are various types of nanostructured lecithin gels, ranging from classic liposomal hydrogels to their modified forms, including transferosomal, ethosomal, phytosomal, and vesicular phospholipid gels (VPG). Several formulations used in anti-aging products have been characterized. Scientists have described liposomes with a particle diameter of 93 nm, which were used to deliver vitamin D3. The liposomes enhanced the therapeutic effect of vitamin D3, ensured the vitamin’s stability, and protected the skin against photoaging by increasing the production of new collagen fibers [34]. Coenzyme Q10 (CoQ10) is a powerful antioxidant used, among other things, to protect against aging. However, its lipophilicity and high molecular weight make its delivery difficult for topical applications. The development of a liposomal formulation of soy phosphatidylcholine and alpha-tocopherol (vitamin E) improved the local bioavailability of CoQ10 and also doubled its accumulation in the skin [34]. Both liposomes and ethosomes have been shown to improve the penetration of substance molecules through the skin. A study by Yücel et al. [16] found ethosomes to be more effective than liposomes in the transdermal delivery of rosmarinic acid. This study was confirmed by measuring the antioxidant activity and inhibitory effect of preparations containing this compound on collagenase and elastase enzymes. The measured size range of ethosome formulations was approximately 138 nm. Nanotechnology enables more effective delivery of active substances to the stratum corneum (SC), and even deeper than traditional methods. This is particularly important for natural antioxidants, whose dietary intake does not ensure sufficient distribution in the skin due to low absorption and rapid metabolism. To protect the epidermis and dermis, they must be delivered locally. Therefore, nanocosmetics should contain stable nanosystems in skin-friendly forms, such as creams or gels, without the need to use invasive methods such as electroporation or microsyringes [25]. Controlled remodeling of the skin’s extracellular matrix (ECM) is essential for its proper development and the maintenance of homeostasis in other organs. ECM remodeling is a hallmark of the pathophysiology of skin aging, wound healing, and certain fatal diseases, including cancer. While numerous aesthetic and medical treatments for skin conditions exist, there is a lack of imaging methods to visualize ECM remodeling in real time, particularly for collagen, which is the most abundant structural component of the skin’s ECM [37].

#### 2.3.3. Fragrance Products

Fragrance is crucial for the perception and effectiveness of products—it influences purchasing decisions and perceived quality. Fragrances are volatile organic compounds that evaporate easily, limiting their shelf life in products. To counteract this, fragrance preservation technologies are used, such as profragrance compounds in which fragrances are chemically bonded to larger molecules and released under specific conditions, and encapsulation, for example, in polymer microcapsules. The latter method not only prolongs the fragrance’s performance but also protects it from external factors (heat, oxygen, light). Various encapsulation systems have been discussed in the literature, including cyclodextrins, inorganic and polymer microcapsules, and polymersomes [38]. Encapsulation involves surrounding the active ingredient with a coating material that protects it from the environment and controls its release. Release can occur under specific conditions or under the influence of external stimuli. Depending on the method, capsules can have different sizes—from nanocapsules (<1 µm) to microcapsules (1–1000 µm). Cyclodextrins are a special case, forming inclusion complexes at the molecular level [38]. Natural and synthetic lipids can self-assemble into structures such as micelles and liposomes, which enable the encapsulation of various molecules—hydrophobic (in micelles) and both hydrophobic and hydrophilic (in liposomes). Due to their biocompatibility, lipids have been widely studied in fragrance encapsulation. For example, phospholipid liposomes with eugenol and lecithin liposomes with a lily scent extended fragrance release. Other studies have shown that liposomes with proteins can be deposited on fabrics (e.g., cotton) and provide controlled release of fragrances, activated by an acidic environment, e.g., sweat. Compared to protein complexes, liposomes released fragrance more slowly but for a longer period. Polymerosomes are synthetic equivalents of liposomes, composed of amphiphilic block copolymers that self-assemble into stable vesicles due to differences in polarity and appropriate block proportions. Their thicker walls provide greater stability than liposomes. Polymerosomes are widely used in medicine, particularly in controlled drug delivery. However, their use for encapsulating fragrances is limited, primarily because fragrances—as hydrophobic or amphiphilic molecules—can only be incorporated into the membrane and not into the vesicle [38]. Beyond regulatory and toxicological considerations, understanding the mechanistic interaction of nanocarriers with the skin barrier is essential for evaluating their real-world performance in cosmetic applications. These safety considerations form a necessary foundation for understanding the mechanistic behavior of nanoscale carriers at the skin barrier, which is discussed in the following section.

### 2.4. Mechanism of Action of Nanoparticles in the Skin

#### 2.4.1. Skin Structure and Its Importance in the Permeation of Nanosomes and Other Nanoparticles

**Figure 7:**
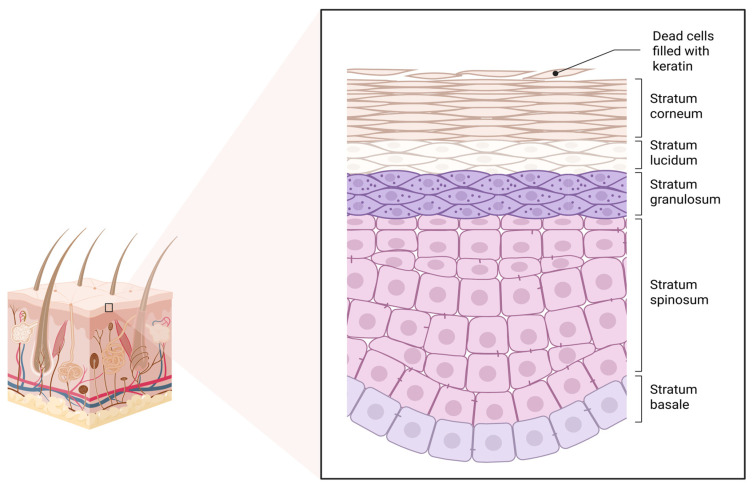
Structure of the skin (figure is original illustrations created using BioRender. Froelich, A. (2026) https://BioRender.com/jb8s1ib; date 13 February 2026).

Skin is directly exposed to many materials, including nanosomes. The skin consists of three layers: the epidermis, the dermis, and the subcutaneous layer [11,12,39,40]. The epidermis consists of a 5 to 20 μm thick layer of keratinocytes and two layers of living cells. The underlying dermis contains hair follicles and sebaceous glands, followed by capillaries. Skin barriers, including keratinocytes and tight junctions, form a tight barrier impermeable to most drug transporters [9]. In addition, melanocytes provide melanin, which absorbs ultraviolet radiation and protects DNA from damage. Langerhans cells, macrophages, and dendritic cells in the dermis act as checkpoints for xenobiotics [9,14]. When developing payloads for transdermal drug delivery, skin barriers must be considered to ensure that nanocarriers can penetrate the skin barriers and reach the systemic circulation. The stratum corneum (SC) is the rate-limiting region for NPs transdermal movement. Furthermore, the physicochemical properties of NPs and the flexural motion of the skin modulate nanoparticle transdermal movement. Nanocarriers have been successfully used to enhance the penetration of therapeutics through the skin and deliver drugs to specific skin zones. Current evidence indicates that nanoscale lipid-based carriers such as nanosomes primarily interact with the outer layers of the skin. Under normal cosmetic use conditions and in the presence of an intact stratum corneum, nanocarriers ≥20 nm penetrate only into the superficial regions of the stratum corneum or accumulate within hair follicles, with minimal or no transport into viable epidermal or dermal layers. Systemic absorption has not been demonstrated for cosmetic formulations and is not expected based on available in vivo human data. Reports describing deeper tissue penetration or vascular uptake generally refer to therapeutic transdermal systems specifically engineered to overcome the skin barrier, often using penetration enhancers, physical disruption techniques, or disease-compromised skin models. Therefore, statements regarding systemic distribution have been reframed to reflect that such outcomes pertain to specialized transdermal drug delivery applications rather than conventional cosmetic products [9,10]. The epidermis, the outermost layer of the skin, constitutes a fundamental physicochemical barrier limiting the penetration of bioactive substances [11,12,40]. It is composed of stratified epithelium dominated by keratinocytes (Figure 7), which undergo continuous differentiation from the basal layer to the surface. These cells are connected by desmosomes, which further restrict the diffusion of substances into the dermis. Keratinization determines the division of the epidermis into four layers: corneum, granular layer, spinous layer, and basal layer, located successively from the surface to the basement membrane [14,39].

The SC consists of dead cells called corneocytes, immersed in an ordered lipid matrix with a “brick and mortar” structure, with characteristic dermal ridges on the surface. SC is considered a lipophilic barrier (containing 15% water in its structure), responsible for most of the protective functions of the epidermis. The remaining, living layers of the epidermis are characterized by a higher water content (approximately 75%) and are more hydrophilic [39]. Disturbance of epidermal homeostasis is closely related to the deterioration of skin health and the pathophysiology of various skin diseases, including ichthyosis, atopic dermatitis, and psoriasis [12]. While keratinocytes constitute over 90% of the epidermis, there is a small percentage of non-epithelial cells found in this layer of skin. These cells include melanocytes, Langerhans cells, and Merkel cells. While keratinocytes are responsible for the mechanical and water barrier function of the epidermis, melanocytes, Langerhans cells, and Merkel cells are responsible for skin pigmentation, immune protection, and sensory function, respectively [39]. Melanocytes originate from the neural crest and produce melanin, which contributes to skin pigmentation. In the basal layer, melanocytes constitute approximately 3% of the cell population [11,40]. The dermis, composed primarily of connective tissue, supports and nourishes the epidermis. Cells constitute approximately 10% of its composition, with fibroblasts—spindle-shaped mesodermal cells that produce collagen and elastin—dominating. Collagen fibers, constituting approximately 70% of the dry weight, provide skin strength, while elastin fibers (<1%) provide elasticity [41,42,43]. Although the ground substance constitutes only 0.2% of the dry weight, it fills most of the dermis. Composed primarily of glycosaminoglycans, water, electrolytes, and plasma proteins, it is responsible for water and electrolyte balance and the structural support of the skin [39,40]. The dermis is divided into two connected layers: the papillary and reticular layers, with no distinct boundary between them. The papillary (upper) layer, composed of loose connective tissue, contains more fibroblasts and loosely arranged collagen and elastin fibers. It forms papillae extending into the epidermis. The reticular (lower) layer is dense connective tissue with fewer fibroblasts and ground substance. Its thick collagen fibers run parallel to the skin surface, while the elastin fibers form irregular structures [39]. Together with the subcutaneous tissue, it provides trophic and mechanical support for the epidermis. Blood vessels and nerve endings are located in these deeper layers of the skin [12]. The subcutaneous tissue connects the skin to the underlying fascia. Composed primarily of adipose tissue, it is well vascularized and participates in the blood supply to and nourishment of the skin [11,12,39]. It also serves as an entry point into the systemic circulation. It contains touch receptors—Pacinian corpuscles—composed of concentrically arranged Schwann cells surrounding a nerve fiber. As loose connective tissue, it serves as a cushion and promotes blood supply to the epidermis and dermis [12].

#### 2.4.2. Mechanism of Nanoparticle Penetration Through the Skin Barrier

**Figure 8:**
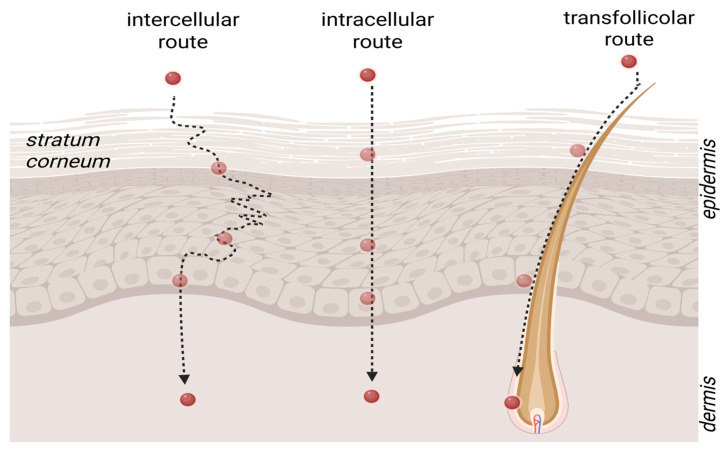
Routes of penetration of substances through the skin (figure is original illustrations created using BioRender. Froelich, A. (2026) https://BioRender.com/jdplg3e; date 13 February 2026).

Penetration of substances through the skin depends on their physicochemical properties. A molecular weight of up to 500 Da (Daltons) is generally considered the upper threshold for effective penetration [14]. Effective drug penetration requires passage through the stratum corneum, the outermost layer of the epidermis. There are essentially two established pathways by which substances can penetrate this barrier [39]. The transepidermal pathway involves the transport of substances across the skin layers and is divided into transcellular and intercellular mechanisms (Figure 8). Available literature indicates that nanoscale carriers exhibit size-dependent behavior: particles ≥ 20–30 nm remain confined to the stratum corneum or follicular openings under normal cosmetic use conditions, whereas particles < 10 nm may traverse partially into the stratum spinosum under experimental or barrier-impaired conditions [20,24,28]. Vesicular systems such as nanosomes (30–100 nm), ethosomes (50–200 nm) and liposomes (>100 nm) therefore differ predictably in their localization profiles, with smaller, highly deformable carriers showing greater surface and follicular distribution but no consistent evidence of viable-layer penetration in intact human skin. It is important to distinguish between the penetration of intact nanocarriers and the penetration of active ingredients released from these systems. Under typical cosmetic use conditions, most nanoscale carriers, including nanosomes, nanoemulsions, and polymeric nanocapsules, remain within the superficial layers of the stratum corneum or accumulate in follicular openings. In many studies, the reported ‘enhanced penetration’ refers primarily to improved solubilization, increased thermodynamic activity, or more favorable partitioning of the released active into skin lipids rather than translocation of the intact nanocarrier. Only specialized transdermal drug delivery systems, often used in therapeutic contexts and relying on chemical or physical penetration enhancers, have demonstrated deeper skin transport of whole nanocarriers. Therefore, when evaluating dermal delivery, it is essential to consider whether observed effects arise from nanoparticle-assisted release near the surface or from true nanocarrier penetration. The mechanistic explanation provided in this section is intended to function as a textual schematic, conceptually outlining the distinct pathways of nanocarrier deposition versus active-compound diffusion. This section provides a mechanistic conceptual summary of nanosome–skin interactions, outlining the key stages of deposition, lipid interaction, active release, and follicular accumulation. This description serves as a textual mechanistic diagram, outlining the sequential steps of nanocarrier behavior at the skin surface, including deposition, follicular accumulation, lipid interaction, active-compound release, and subsequent molecular diffusion.

Transcellular penetration involves the direct passage of substances through cells, which favors hydrophobic compounds due to the lipid nature of the membranes. In the intercellular mechanism, substances move through the lipophilic extracellular matrix between cells, making this pathway dominant for hydrophobic drugs. The transadnexal pathway involves drug penetration through hair follicles and sebaceous glands. It facilitates the transport of polar, ionizable molecules and macromolecules that are difficult to cross the stratum corneum. Skin appendages create alternative pathways, favoring the absorption of larger and more complex compounds [25,39]. Although the dominant mechanism of transepidermal transport remains a matter of debate, it is generally accepted that hydrophobic compounds prefer the intercellular route, while hydrophilic compounds prefer the transcellular route. Beyond basic transepidermal pathways, the penetration behavior of nanocarriers is governed by their physicochemical and biomechanical properties as well as by the characteristics of the formulation vehicle. Vesicular systems differ in deformability: conventional liposomes exhibit limited flexibility, whereas ethosomes, enriched with ethanol, display increased membrane fluidity, and transferosomes possess ultradeformable bilayers capable of temporarily adapting their shape to pass through narrow intercellular spaces of the stratum corneum [25,44]. Surface charge and hydrophilicity further modulate interactions with skin lipids and proteins; however, their influence is highly context-dependent and cannot be generalized across nanocarrier types. PEGylation enhances steric stabilization and prolongs residence on the skin surface but does not typically facilitate deeper penetration. The hydration state of the stratum corneum plays a critical role: occlusive formulations increase water content, widen intercellular lipid domains, and enhance diffusion of released actives. Vehicle composition—particularly solvent polarity, volatility, and rheological behavior—affects thermodynamic activity, nanoparticle aggregation, and partitioning into the skin barrier. In addition, hair follicles act as preferential entry sites for nanocarriers, serving as a reservoir enabling sustained release and prolonged retention within the pilosebaceous unit. Taken together, these mechanistic factors illustrate that nanocarrier penetration is not determined by size alone but results from a complex interplay between vesicle properties, formulation matrix, and skin physiology.

A deeper mechanistic understanding of nanocarrier penetration requires consideration of vesicle deformability, steric stabilization mechanisms, vehicle rheology, and occlusion-mediated hydration effects [25]. Ultradeformable vesicles such as transferosomes possess highly flexible bilayers enriched with edge activators (e.g., surfactants), allowing them to transiently adapt their shape and pass through intercellular lipid channels that are narrower than their unperturbed diameter—an ability not shared by conventional liposomes, which display limited membrane elasticity. PEGylation, frequently used to enhance colloidal stability, provides steric repulsion between vesicles and surrounding biomolecules; however, this stabilizing effect often reduces interactions with stratum corneum lipids, thereby diminishing the likelihood of deep penetration and favoring prolonged surface residence instead [34]. Vehicle rheology and interfacial properties further modulate penetration efficiency by influencing nanocarrier spreading, film formation, retention on the skin surface, and the degree to which particles partition into the lipid matrix. Low-viscosity vehicles enhance initial contact but may promote aggregation or rapid evaporation, while structured or gel-based systems can modulate residence time and control the spatial distribution of vesicles [45]. Finally, occlusion plays a central role in altering barrier function: occlusive formulations increase hydration of the stratum corneum, expand intercellular lipid spacing, and reduce tortuosity of diffusion pathways, thereby enhancing the mobility of released actives and, in certain cases, facilitating the passage of highly deformable carriers [46]. Together, these parameters demonstrate that nanocarrier penetration is governed not only by particle size but by a complex interplay of biomechanical adaptability, interfacial stabilization, vehicle structure, and hydration-modulated barrier dynamics.

Lipophilic substances penetrate through the lipid layers present between corneocytes, whereas hydrophilic substances utilize the aqueous pores formed by the hydrophilic regions of lipids and the corneocytes themselves. An alternative, although limited in surface area, route is the adnexal pathway, which includes glands and hair follicles [14]. Passive skin penetration primarily affects compounds with low molecular weight and a moderate oil/water partition coefficient. Although lipophilic compounds penetrate the stratum corneum more readily, their further diffusion into the skin can be limited [39]. In addition to the physicochemical properties of the molecules, skin penetration is influenced by factors such as skin metabolism, the location and condition of the tissue at the application site, and the presence of a carrier. Surface charge is one of several physicochemical parameters that can influence interactions between nanocarriers and the stratum corneum; however, its effect on penetration is highly context-dependent. Some studies report enhanced interaction of negatively charged or neutral vesicles with skin lipids, whereas others show improved adhesion or retention of positively charged systems due to electrostatic interactions with the slightly negatively charged skin surface. Overall, the impact of charge cannot be generalized, as penetration behavior depends on a combination of factors, including particle size, composition, degree of deformability, surface chemistry, formulation vehicle, and the condition of the skin barrier. Therefore, charge should be considered as a contributing parameter rather than a standalone predictor of dermal penetration. Product formulation is also crucial in cosmetics, as it can alter polarity, the lipophilic–hydrophilic ratio, and the structure of the epidermal barrier. Rheological properties, such as viscosity and phase behavior, are also crucial, determining the bioavailability of active ingredients [25,45,46]. To understand the effects of niosomes on the skin, their interactions with the SC were investigated using microscopic assays. As it showed, the concentration of vesicular components is higher at or around the surface of the SC and gradually decreases in the inner region of the stratum corneum [47]. It is assumed that niosomes associate with native SC lipids. Although some images of vesicular components have been reported even in the inner sections of the SC, it is unclear whether these represent intact niosomes that have migrated from the skin surface or whether uncontrolled vesicle renewal occurs as the SC becomes more hydrated. They demonstrated that niosomes were significantly more effective in delivering enoxacin than liposomes or a simple solution of the active ingredient. The authors of the above study observed that niosomes can penetrate the SC of the skin, especially when its barrier function is impaired, allowing the delivery of even larger molecules. Under normal cosmetic use conditions and intact skin, these effects correspond primarily to increased hydration, lipid interaction, and enhanced release of the encapsulated compound, rather than migration of intact niosomes into deeper layers. Niosomes demonstrate potential for transdermal drug delivery, especially in cases where the skin barrier is impaired, which could be used in topical therapy for various dermatological conditions [47]. The way in which the physicochemical properties of NPs determine penetration, systemic translocation, and toxicity has been extensively studied. It is assumed that these properties, such as shape, chemical composition, stability, surface area, and charge, have a decisive influence on interaction with the skin [34]. To achieve an effective therapeutic effect, nanocarriers and nanoparticles must cross the SC barrier. Transdermal delivery of particularly hydrophilic molecules is hindered by the epidermal lipid layer. Various technologies, such as micropuncture, cavitation ultrasound, microdermabrasion, electroporation, and thermal ablation, are used to create perforations in the SC [34]. Another interesting solution is the effect on SC exfoliation, which can be observed, for example, in the use of PLGA (poly-(lactide-co-glycolic acid)), a biodegradable and biocompatible polymer that has been used in studies involving a variety of biological materials. PLGA-based particles are used in the formulation of drug delivery systems. PLGA initially decomposes through hydrolysis, resulting in the formation of lactic acid and glycolic acid monomers. Lactic acid and glycolic acid are α-hydroxy acids that are effective cosmetic ingredients. They reduce the strength of the SC by exfoliating the outermost layer of corneocytes, which reduces the visibility of wrinkles on the skin, but also affects the ability to penetrate the skin. Reports describing the penetration of PLGA nanospheres of approximately 200 nm into deeper skin layers largely stem from studies conducted on animal models, ex vivo tissues, or barrier-disrupted skin, or from experiments employing chemical or physical penetration enhancers. Under normal cosmetic use conditions and in intact human skin, nanoparticles of this size—including PLGA systems—are generally retained within the stratum corneum or follicular openings, with negligible transport into the viable epidermis or dermis. Therefore, any observed deeper localization should be interpreted within the context of the experimental model and should not be extrapolated to routine application on healthy human skin [48]. These mechanistic insights provide the foundation for evaluating broader challenges and future directions in the development, assessment, and responsible use of nanoscale cosmetic delivery systems.

### 2.5. Challenges and Prospects for the Use of Nanosomes and Other Nanoparticles in Modern Cosmetology

**Figure 9:**
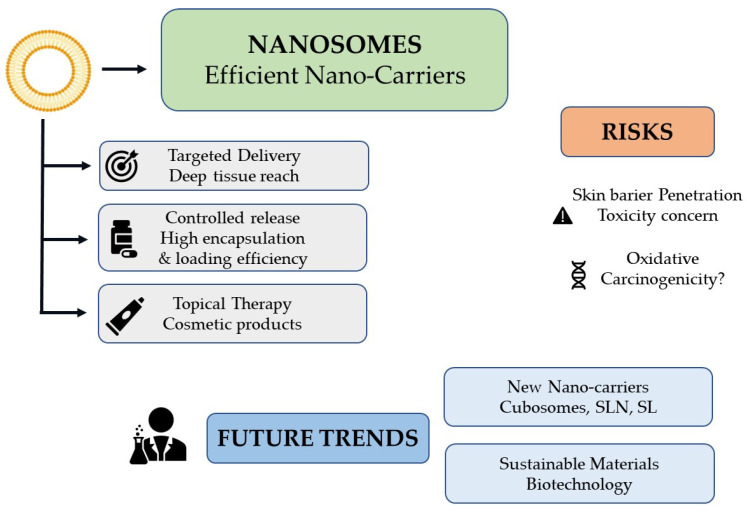
Role of nanosomes in modern cosmetology: benefits, risks, and perspectives (figure prepared by authors).

Despite their functional advantages, nanoscale carriers present several trade-offs. Nanosomes offer favorable biocompatibility but are more sensitive to oxidative degradation than polymeric systems. Ethosomes provide enhanced flexibility but require high ethanol content, limiting compatibility with sensitive actives. Transferosomes exhibit superior deformability yet lack long-term stability and scalability for mass-market cosmetic production [35]. Nanoemulsions are highly scalable and cost-efficient but cannot transport intact droplets across the skin barrier. Polymeric nanocapsules offer exceptional stability and EE but involve more complex and costly manufacturing procedures [27,32]. These limitations highlight the need to balance performance, cost, sustainability, and regulatory feasibility when selecting nanocarriers for cosmetic applications. Nanosomes constitute an effective system for localized delivery of active ingredients within cosmetic formulations. In cosmetic applications, their role is limited to enhancing surface deposition, formulation stability, and controlled release of actives within the upper layers of the skin. Importantly, this mode of action does not involve systemic distribution or transport to internal organs, which remains outside the scope of cosmetic use conditions [9,10,24]. Despite their many advantages, nanocosmetics also raise serious safety concerns, mainly due to their ability to penetrate the skin barrier, which can lead to toxicity, oxidative stress, and, in extreme cases, carcinogenicity [30,49]. Therefore, it is necessary to conduct long-term toxicological studies and implement strict regulations governing the composition, production, storage, and marketing of cosmetics containing NPs [3]. NanoTech is currently one of the most promising and dynamically developing tools used in cosmetology, cosmeceuticals, dermatology, and biomedical applications. The use of modern carrier systems such as liposomes, nanoliposomes, ethosomes, nanoemulsions, and niosomes allows for significant improvements in the effectiveness of cosmetic formulations, increasing their stability, bioavailability, and comfort of use. These products have gained widespread consumer acceptance worldwide and have become an indispensable element of daily care [30,49]. In addition to conventional formulation challenges, emerging technologies contribute new opportunities and considerations for next-generation nano-enabled cosmetic systems. Recent advancements in additive manufacturing (3D printing) provide new opportunities for integrating nanocarrier systems into structured topical formulations. 3D printing enables the fabrication of customized hydrogel matrices, solid dispersions, and nano-structured scaffolds with precise control over porosity, geometry, and spatial distribution of active ingredients. This technology allows nanocarriers such as nanosomes, nanoemulsions, and polymeric nanocapsules to be incorporated directly into printed hydrogels or solid polymer networks, potentially enhancing stability and enabling programmable release profiles. Additionally, the capacity of 3D bioprinters to process biocompatible and temperature-sensitive materials makes them suitable for embedding lipid-based nanocarriers without compromising their structural integrity. Although the application of 3D printing in cosmetics is still emerging, early studies suggest that it could facilitate the development of personalized skin-care products and targeted delivery systems, as well as support the fabrication of bio-inspired nano-structured materials. Therefore, 3D printing represents a promising complementary technology for future nano-enabled cosmetic formulations. The cosmetics sector is developing rapidly, with products being developed by both large and local companies worldwide. The introduction of new advances and active-ingredient delivery systems is expanding the cosmetics sector and increasing its market share (Figure 9). Nowadays, cosmetics are crucial to our daily activities, and the use of NanoTech in cosmetic products has further improved their acceptance among consumers worldwide. Currently, new biodegradable nanocarriers such as ethosomes, niosomes, cubosomes, solid lipid nanoparticles, and liposomes are being used to formulate a variety of cosmetics with excellent properties [35]. Nanocosmetics can deliver nanoformulations through human skin via various routes, providing multiple applications, e.g., moisturizing, sun protection, wrinkle reduction, etc. While these nanomaterials are achieving remarkable market value and consumer interest, there are significant concerns regarding their toxicity and safety for humans, which require more in-depth research and analysis [35,49].

A key consideration in translating nanoscale delivery systems from laboratory research to commercial cosmetic formulations is the set of scale-up challenges that arise when processes are transferred from controlled bench-top conditions to industrial production. Methods such as ultrasonication, high-pressure homogenization, or solvent-based vesicle formation often show good laboratory reproducibility but may suffer from batch-to-batch variability, increased sensitivity to raw-material quality, or higher production costs at manufacturing scale [3,17]. Stability is also a major constraint: nanosomes and conventional liposomes are prone to oxidation or aggregation over time, whereas highly deformable vesicles such as transferosomes may lose structural integrity under shear forces encountered during scale-up [25]. From a regulatory perspective, commercialization is further limited by EU and FDA requirements for nanoform characterization, nano-specific labeling, safety substantiation, and nanoform-by-nanoform documentation under frameworks such as Regulation (EC) 1223/2009 and REACH, which substantially increase development timelines and data generation needs [3,7,8]. Beyond regulatory hurdles, consumer acceptance represents an additional translation barrier: public perception of ‘nanotechnology’ is often shaped by misconceptions about nanoparticle safety, requiring careful communication, transparent labeling, and formulation choices that avoid components (e.g., high ethanol content or strongly occlusive systems) perceived as undesirable in cosmetics [3,15]. Formulators must also navigate practical constraints such as compatibility of nanocarriers with emulsifiers, preservatives, fragrances, pH ranges, and packaging materials [3,21,34]. These combined factors illustrate that the successful transition of nanocarrier technologies into mainstream cosmetic products requires not only scientific optimization but also regulatory, manufacturing, and consumer-oriented considerations. In addition to regulatory and methodological challenges, an important barrier to the broader implementation of nanocarrier-based cosmetic products is consumer perception and understanding of nanotechnology. Although lipid-based and polymeric nanosystems used in cosmetics demonstrate favorable safety profiles and undergo regulatory evaluation, public concerns often arise from misconceptions about the term ‘nanoparticles’ and the assumption that all nanoscale materials exhibit similar risks. Therefore, clear communication and education—through transparent labeling, informational materials, professional seminars, and scientifically grounded media outreach—are essential to increase consumer awareness and support informed decision-making. Improving public understanding directly addresses one of the key challenges for the field, as social acceptance significantly influences the successful adoption and responsible introduction of nanocarrier-enabled formulations. Only a comprehensive approach, combining technological development with appropriate regulations and education, can ensure the safe and effective use of nanocosmetics in modern skincare. The nanomarket for cosmetic products began in the late 1980s. The first cosmetic product containing liposomes to hit the market was the anti-aging “Capture,” introduced by Christian Dior in 1986. This was followed in 1998 by “Plentitude Revitalift” (L’Oréal), an anti-aging cream containing polymer nanocapsules for the delivery of active ingredients, such as retinol. Today, 40 years later, the use of NanoTech in cosmetic products sold worldwide is so widespread that current attempts to categorize them are quickly becoming obsolete [49,50]. Cosmetics production is currently harnessed by nanotechnology, but the future of the industry will largely depend on technological advances offered by omics sciences, which, combined with big data analysis and machine learning approaches, will allow us to better assess biological responses to specific cosmetic formulations and bioactive compounds at the cellular and tissue levels. This scenario is well integrated with the concept of “cosmeceuticals,” the idea that a cosmetic has scientifically proven preventive or therapeutic effects. This claim is becoming increasingly popular thanks to its dissemination on social media and has a direct impact on consumers and market demand. Consumers are now advocating for the inclusion of new bioactive or functional ingredients in cosmetic products to promote cellular revitalization by introducing anti-aging and antioxidant properties. Biodiversity is a new (or even old) source of these natural and sustainable compounds. In fact, significant research efforts are currently underway in the bioprospecting and evaluation of natural ingredients regarding their mechanisms of action on the skin [14]. Natural products, whether derived from plants, animals, or microorganisms, offer an unparalleled range of chemical diversity, providing a key foundation for drug discovery and development. Their unique molecular structures open the door to new drug candidates with diverse mechanisms of action, providing promising solutions for complex diseases and drug-resistant pathogens. However, the use of natural products faces various challenges due to their inherent physical and chemical properties. Therefore, proactive solutions are essential, and drug delivery systems have emerged as a key approach. NanoTech, as the cornerstone of nanoscale DDSs, holds enormous promise in this regard. Remarkably, the particle size of these systems, a key factor, significantly influences various aspects of drug delivery, including their efficacy, biodistribution, and overall therapeutic impact [19]. In the context of healthy and sustainable cosmetics, nanobiotechnology can play a key role for two main reasons. First, NanoTech enables the design of biomimetic NPs capable of transporting natural metabolites directly to target cells, which is crucial for achieving the desired biological effects. Furthermore, the structural properties of NPs—such as size, shape, chemical composition, and surface charge—can be precisely modified to increase penetration efficiency through the epidermal and dermal layers [14,39]. A second important aspect is the use of biotechnology for the biosynthesis of excipients used in cosmetic formulations. In response to the growing demand for natural and organic products, there is also a growing need to eliminate preservatives, dyes, and synthetic additives such as silicones. In this context, biotechnology supports the development of biodegradable materials, such as bioplastics, which can replace conventional synthetic raw materials, contributing to greater sustainability in production processes [14].

The future development of cosmetic nanocarriers will depend strongly on overcoming several scientific and regulatory challenges that currently limit their full implementation. Despite extensive progress, significant gaps remain in standardized characterization protocols, long-term safety evaluation, and harmonized methodologies for assessing skin penetration. Existing studies are often difficult to compare due to variability in experimental conditions, nanocarrier composition, particle size, and the choice of in vitro or ex vivo models. This highlights the need for universally accepted analytical standards, particularly for assessing particle stability, aggregation behavior, and release kinetics under conditions representative of cosmetic use. Another critical area concerns the long-term toxicological profile of nanosystems. While most lipid-based nanocarriers, including nanosomes, display favorable biocompatibility, comprehensive data on chronic exposure, cumulative effects, and interactions with compromised skin remain limited. From a regulatory perspective, the increasing complexity of nano-enabled formulations underscores the importance of transparent risk assessment frameworks and coherent guidelines that clearly differentiate between dermal and inhalation exposure routes. The advancement of computational toxicology, predictive modeling, and high-resolution imaging techniques will be instrumental in strengthening safety evaluations. Looking ahead, the cosmetics industry is expected to benefit from innovations in biodegradable polymers, green nanotechnology, and precision-engineered lipid systems that offer improved stability and targeted delivery. Emerging technologies such as 3D printing, bio-inspired nanostructures, and advanced encapsulation strategies will enable greater personalization and functional sophistication of formulations. Nanosomes, in particular, are well-positioned to remain central to the field due to their favorable encapsulation efficiency, compatibility with skin lipids, and regulatory acceptance. However, their continued advancement will depend on integrating improved manufacturing scalability, enhanced stability profiles, and robust safety documentation. Taken together, these considerations suggest that while nanocarriers offer substantial promise for next-generation cosmetic products, their responsible development will require a coordinated effort across formulation science, analytical methodology, risk assessment, and regulatory policy. Addressing these interconnected challenges will be key to unlocking the full potential of nanotechnology-enabled skincare.

An additional challenge associated with the introduction of nanocarrier-based cosmetic formulations is the role of consumer perception and public understanding of nanotechnology. Although many nano-enabled ingredients used in cosmetics—particularly lipid-based systems such as nanosomes—have favorable safety profiles supported by regulatory evaluations, misconceptions about the term ‘nanoparticles’ may lead to unnecessary concern among consumers. The distinction between dermal exposure, which is generally considered safe for many nanomaterials used in cosmetics, and inhalation risks associated with sprays or powders is often poorly understood outside scientific and regulatory contexts. Therefore, transparent communication and consumer education, supported by accessible informational materials, professional seminars, and science-based media outreach, are essential for enabling informed product choices. Increasing public awareness of how different nanocarriers function and how they are regulated can help reduce hesitation and support the responsible implementation of safe, evidence-based nano-cosmetic technologies.

## 3. Conclusions

Nanotechnology plays an increasingly important role in cosmetic formulation design, offering nanoscale carriers capable of improving solubility, stability, and targeted delivery of active ingredients. Among these systems, lipid-based nanosomes stand out due to their biocompatibility, structural similarity to biological membranes, and favorable encapsulation capacity. Although nanocarriers can enhance the local availability of cosmetic actives, evidence consistently shows that intact nanoparticles ≥ 20 nm remain confined to the stratum corneum or hair follicles when applied to healthy human skin. Therefore, their benefits arise primarily from their improved surface deposition, controlled release, and enhanced partitioning rather than systemic penetration. Safety evaluation remains essential, and current regulatory frameworks—including EU Regulation 1223/2009, SCCS opinions, FDA guidance, and OECD test guidelines—provide structured methodologies for assessing dermal exposure, irritation, sensitization, and long-term risks. Future progress will depend on harmonized characterization standards, transparent risk communication, and continued development of sustainable, biodegradable nanocarriers. Overall, nanosomes and related nanosystems represent a promising and expanding platform in modern cosmetology, provided that innovation is accompanied by robust safety assessment and responsible regulatory oversight.

## Figures and Tables

**Table 1:** Comparative characteristics of major nanocarriers used in cosmetic formulations.

Nanocarrier Type	Typical Size	Composition	Encapsulation Efficiency	Penetration Behavior	Stability	Regulatory/Safety Notes	Commercial Maturity/TRL
Nanosomes/nanoliposomes	30–100 nm	Phospholipid bilayers	High (hydro + lipophilic)	SC surface + follicles; no deep penetration	Moderate; sensitive to oxidation	Excellent biocompatibility; strong regulatory acceptance	High—widely used in cosmetic formulations; industrial processes well established
Liposomes (conventional)	100–300 nm	Phospholipids	High (hydrophilic)	Limited penetration	Moderate	Safe; widely used	High—long history of commercial use in cosmetics and dermatology
Ethosomes	50–200 nm	Phospholipids + ethanol	High	Higher deformability; enhanced SC permeation	Lower stability (ethanol)	Safe; formulation constraints due to alcohol	Medium—promising technology but with formulation constraints due to ethanol content
Transferosomes	80–200 nm	Phospholipids + edge activators	Very high	Ultradeformable; intercellular transport	Moderate	Mostly pharmaceutical; less common in cosmetics	Low–Medium—high mechanistic potential; limited widespread commercialization due to stability issues
Niosomes	100–300 nm	Non-ionic surfactants	High	Variable; depends on surfactant grade	High	Surfactants may irritate	Medium–High—inexpensive materials and scalable production; increasing cosmetic use
Nanoemulsions	20–200 nm	Oil droplets + surfactants	High for lipophilic	Do not penetrate intact; enhance release & partitioning	High kinetic stability	Safe dermally; avoid inhalation forms	Very High—one of the most mature, scalable and commercially adopted nanocarrier systems
Polymeric nanocapsules	50–300 nm	Biodegradable polymer shell + oily core	Very high	Follicular targeting; controlled release	Very high	Depends on polymer biodegradability	Medium—strong scientific maturity; moderate commercial adoption due to production complexity
Polymerosomes	100–300 nm	Block copolymers	Very high	High stability; limited penetration	Very high	Mostly pharma; limited cosmetic use	Low—robust performance but limited commercial readiness in cosmetics

**Table 2:** Examples of nanoparticle’s benefits.

Benefits of Nanoparticles			
Better Properties	Increased Operational Efficiency	Extended Product Stability	Specialized Use
Better texture of the product	Better absorption through the skin	Longer shelf life of cosmetics	Effective antioxidant delivery—encapsulation and targeted distribution
Smoothenes	More effective penetration of active ingredients	Protection against the decomposition of active substances	Transparent and effective sunscreens containing zinc oxide and titanium dioxide (no white cast)
Increased transparency of formulation	Optimized distribution of active substances	Greater stability of product ingredients	

## Data Availability

Publicly available publications were analyzed in this study. All used sources are included in the list of references.
